# Feeding microbes to feed the Gut: inulin reprograms intestinal epithelial metabolism and proliferation through HIF1α

**DOI:** 10.1080/19490976.2026.2644684

**Published:** 2026-03-18

**Authors:** Raphael R. Fagundes, Sean P. Colgan

**Affiliations:** a Department of Cell & Chemical Biology, Leiden University Medical Center (LUMC), Leiden, The Netherlands; b Division of Gastroenterology and Hepatology, Department of Medicine, Mucosal Inflammation Program and University of Colorado, Aurora, CO, USA; c Department of Medicine, Rocky Mountain VA Health Care System, Aurora, CO, USA

**Keywords:** Hypoxia, dietary fiber, HIF-1, inulin, intestinal epithelium

## Abstract

Indigestible dietary fibers shape intestinal mucosal physiology, yet the mechanisms linking fiber-derived microbial activity to epithelial remodeling remain incompletely understood. In their recent study, Ribeiro Castro et al. revealed that the prebiotic inulin induces reprogramming of intestinal epithelial metabolism and proliferation through microbiota-dependent hypoxia and epithelial HIF1α activation. In this commentary, we discuss their findings and highlight the emerging concept that microbial fermentation and oxygen concentrations act as structured physiological signals that guide intestinal epithelial differentiation and crypt–villus dynamics. We further explore how these findings intersect with prior work on SCFA metabolism, butyrate-mediated ISC inhibition, and fructose-driven epithelial growth, and we discuss open questions regarding downstream HIF1α programs, niche accessibility, and immune‒epithelial crosstalk. Understanding how HIF1α calibrates this balance will be essential for safely leveraging prebiotics and microbiome-targeted interventions that promote mucosal health.

The intestinal epithelium consists of a single layer of cells that provides a physical barrier between the subepithelial mucosa and the luminal contents of the gut. In this context, intestinal epithelial cells (IECs) maintain a constant host‒microbe crosstalk that allows for the absorption of nutrients, physiological adaptations, immune cell priming, as well as the sensing of danger signals that originate from the lumen. This communication is partially accounted for by the activity of pattern recognition receptors, as well as by the metabolism of luminal and microbial-derived small molecules by IECs. Of particular importance, short-chain fatty acids (SCFAs) have been widely studied over the past two decades and are well-recognized to regulate energy metabolism in IECs, which in turn has been linked to improved mucus production, barrier function, and mucosal healing. Indigestible dietary fibers, such as inulin, can deliver benefits by feeding microbial fermentation and, as such, function as prebiotics. Although still incompletely understood, inulin has been identified for its anti-inflammatory, barrier-protective, and immune-modulatory capabilities in a variety of *in vivo* and *in vitro* studies. Recent studies have increasingly highlighted that the beneficial influences of inulin are largely dependent on the composition and metabolic activity of the gut microbiota. Inulin serves as a substrate for bacterial fermentation, leading to the production of SCFAs and other metabolites that can profoundly reshape the intestinal environment. However, the molecular and metabolic mechanisms linking inulin-derived microbial activity to epithelial remodeling have remained incompletely understood.

In this issue of *Gut Microbes*, Ribeiro Castro et al. provided important new insight into this question by uncovering how inulin reprograms intestinal epithelial metabolism and proliferation through HIF1α-dependent mechanisms.^1^ Using a combination of single-cell transcriptomics, spatial profiling by immunohistochemistry, and organoid validation, the authors demonstrated that inulin ingestion induced a microbiota-dependent remodeling of intestinal epithelial cell populations. Notably, this response involves transcriptional reprogramming of metabolic pathways across distinct epithelial compartments, showing decreased gene expression of pathways involved in oxidative phosphorylation (OxPhos) signatures in intestinal stem cells (ISCs) and transit-amplifying cells but enhanced expression of pathways involved in mitochondrial metabolism in differentiated enterocytes. These data suggest that fiber-driven microbial metabolism may modulate metabolic substrate usage across the intestinal mucosa, reshaping not only epithelial physiology but also proliferative dynamics. The authors propose that this metabolic remodeling is initiated by microbiota-derived hypoxia, which stabilizes HIF1α in intestinal epithelial cells. This mechanism aligns with recent work showing that bacterial fermentation and SCFA oxidation can locally reduce oxygen tension in the gut epithelium.^2-4^ Together, these findings extend the concept of microbiota-induced epithelial hypoxia from a metabolic by-product to a structured physiological signal capable of reprogramming intestinal epithelial metabolism and differentiation.

A particularly intriguing aspect of the study is the observation that epithelial-specific loss of HIF1α led to increased epithelial proliferation in inulin-fed mice, accompanied by longer colons and crypts. This paradoxical effect suggests that HIF1α may act as a metabolic “handbrake”, restraining excessive mitochondrial respiration and limiting epithelial expansion under nutrient-rich or hypoxic conditions. Such a model resonates with evidence that HIF1α activation favors glycolytic metabolism and preserves intestinal homeostasis.^5-7^ In this framework, HIF1α acts not only as a hypoxia sensor but also as a metabolic gatekeeper that balances epithelial growth and energy expenditure. While we do not know the exact molecular link between HIF1α and attenuated epithelial proliferation, a plausible explanation may lie in the epigenetic control of proliferating stem cells of the intestinal crypt. For instance, it is known that HIF and histone deacetylases can work in concert to regulate gene transcription.^8^ Work in intestinal immune cells has shown that HIF1-driven glycolytic flux controls acetyl-CoA and specific histone H3K27 acetylation, linking the metabolic state to chromatin access and proliferation/function.^9^ By analogy, epithelial HIF1α may tune histone acetylation via acetyl-CoA pools; loss of HIF1α could change acetyl-CoA dynamics and SCFA-derived acetylation, favoring a more proliferative chromatin state in crypt cells under high-fiber conditions. Therefore, in the absence of HIF1α and the presence of prominent histone deacetylase inhibition (i.e. provided by butyrate), epithelial cells likely process SCFAs and other microbiota-derived metabolites differently, changing the balance of energy substrates and epigenetically active metabolites available to stem and transit-amplifying cells, which permits or enhances their proliferation under the influence of substrates such as inulin.

The transcriptional data presented by Ribeiro Castro et al. highlight striking cell-type-specific metabolic shifts. While OxPhos genes were downregulated in stem- and transit-amplifying compartments, they were upregulated in mature enterocytes. This divergence raises an important mechanistic question: does reduced OxPhos in ISCs reflect a return to a more glycolytic, stem-like metabolism, or does it signal a protective adaptation to a hypoxic environment? Conversely, increased mitochondrial activity in differentiated cells could promote local oxygen consumption, thereby maintaining the crypt niche in a relatively hypoxic state while simultaneously limiting the availability of energetic substrates for neighboring stem cells and influencing their metabolic state. These dynamic oxygen and substrate gradients may thus reinforce compartmental specialization, a concept supported by prior work showing that epithelial metabolism itself contributes to the establishment of mucosal oxygen and gut-derived metabolite profiles.^3^^,^^10^ Further studies will be required to determine which downstream programs of HIF1α mediate these epithelial changes. Beyond its canonical role in glycolytic gene regulation, HIF1α has been shown to influence lipid and amino acid metabolism, mitochondrial organization, and redox balance.^11^^,^^12^ How these pathways intersect to regulate ISC maintenance and differentiation in response to dietary fiber remains an exciting area for exploration.

The work by Ribeiro Castro et al. builds upon a growing body of literature linking microbial metabolites to epithelial oxygen dynamics. The finding that the colon of inulin-fed mice became more hypoxic than that of mice fed a conventional diet supports prior observations that bacterial fermentation lowers luminal oxygen concentrations, at least in part through SCFA (beta-)oxidation. However, the downstream consequences of this localized hypoxia appear complex. When HIF1α was deleted specifically in IECs, inulin induced an even greater proliferative response, suggesting that HIF1α restrains, rather than promotes, IEC proliferation. This observation raises intriguing parallels and contrasts with prior studies.

For example, Kaiko et al. ^10^ demonstrated that butyrate inhibited ISC proliferation through a mechanism that relies on the architecture of the intestinal crypt.^10^ It has been demonstrated, for instance, that a natural metabolite gradient stemming from the top of the villus to the bottom of the crypt results in the rapid consumption of butyrate as fuel by differentiated IECs, thereby minimizing exposure of the crypt to butyrate. In crypt-less organisms (e.g. zebra fish) or intestinal injury (e.g. DSS colitis) this gradient is disrupted by the loss of villus architecture and ISCs are unnaturally exposed to metabolites such as butyrate. Moreover, we previously reported that butyrate alone was insufficient to drive epithelial proliferation, whereas inulin-derived fructose could sustain IEC growth.^13^^,^^14^ These findings suggest that multiple microbial metabolites, beyond butyrate, contribute to the proliferative response to inulin. Whether changes in fructose transporter expression, butyrate receptor signaling, or purine cross-feeding pathways^15^ underlie these observations remains to be determined.

The idea that HIF1α acts as a metabolic brake on proliferation fits with its well-established role as a regulator of glycolysis and mitochondrial metabolism. Ribeiro Castro et al. proposed that loss of HIF1α permits enhanced mitochondrial respiration and fatty acid oxidation, increasing the consumption of available butyrate by differentiated enterocytes. In the context with growing evidence, this, in turn, could be proposed to reduce butyrate accessibility to the crypt base, relieving the inhibitory pressure on ISCs and promoting proliferation. Such a model integrates metabolic and spatial aspects of the epithelial landscape, suggesting that dietary fiber-induced HIF1α activity maintains an oxygen and metabolite gradient that preserves crypt homeostasis. Without HIF1α, this balance may shift toward excessive oxidative metabolism and epithelial expansion. Nevertheless, several mechanistic aspects remain open: for example, it will be essential to assess whether inulin or HIF1α deletion alters the proportions of ISC, transit amplifying, and enterocyte populations. Quantitative lineage tracing using Lgr5⁺ markers, coupled with metabolic flux analysis, could help clarify whether changes in proliferation are due to intrinsic ISC activation or to altered niche accessibility.

The physiological implications of these findings extend beyond metabolism. While inulin has previously been shown to improve barrier function and protect against inflammation in some settings,^16-20^ this can vary depending on the microbial composition and experimental context. Multiple studies have demonstrated that inulin and other fermentable fibers can exacerbate experimental colitis by aggravating epithelial damage, promoting inflammatory innate lymphoid cells (ILC2) or MyD88/IL‑18-dependent responses, and worsening disease severity in both maternal and adult dietary settings.^21-25^ These reports collectively indicate that inulin can exacerbate epithelial injury and inflammation in susceptible hosts, but the precise dietary, microbial, and host factors that determine harm versus benefit remain incompletely defined, and further mechanistic and translational studies are clearly needed.

HIF1α signaling has been implicated in maintaining epithelial barrier integrity and modulating immune tone. Whether HIF1α loss compromises or enhances epithelial resilience under inflammatory stress remains unclear. Functional protection assays in disease models will be crucial to determine whether the observed epithelial proliferation translates into better or worse mucosal outcomes. Moreover, the immune system represents an important missing link in this metabolic loop. Previously, the proliferative influence of an inulin-supplemented diet on IEC proliferation was partially explained via the cross-communication with mucosal γδ T lymphocytes and *IL22* expression in a similar mouse model.^20^ Furthermore, HIF1α-dependent *IL18* expression by epithelial cells is known to sustain mucosal homeostasis and influence immune cell recruitment.^6^^,^^26-28^ It will be informative to assess whether inulin-induced hypoxia impacts cytokines such as IL-22 and IL-18, bridging epithelial and immune responses. Similarly, the potential impact of this inulin-HIF1α axis on other immune cell subsets, particularly regulatory and effector populations that respond to an inulin diet such as T cells and innate lymphoid cells,^20^^,^^22^^,^^29^ warrant further investigation.

## Future perspectives and conclusion

Together, these findings open an exciting window into how dietary components, microbial metabolism, and epithelial hypoxia intersect to shape intestinal homeostasis. Future work should aim to map the spatial distribution of key metabolites, such as butyrate, purines and fructose, along the crypt axis, and to link these gradients with ISC dynamics over time. Complementary metabolic approaches, including Seahorse analysis and stable isotope tracing, could directly test whether an inulin diet or HIF1α deletion alters intestinal epithelial fuel preference. In parallel, exploring how inulin reshapes the microbial cross-feeding of purines and other metabolites may reveal additional regulatory pathways influencing epithelial repair. From a broader perspective, this study emphasizes that diet-induced metabolic rewiring can be both protective and risky. While microbial fermentation products like SCFAs support barrier maintenance and healing, excessive epithelial proliferation may compromise stability or increase susceptibility to tumorigenic changes if unchecked. Understanding how HIF1α calibrates this balance between regeneration and restraint will be essential for designing safe microbiome-targeted dietary interventions.

Ribeiro Castro et al. provided a compelling demonstration that prebiotics, such as inulin, have the capacity to reprogram the intestinal epithelium through HIF1α-dependent mechanisms. These findings highlight a novel axis linking high-fiber diet, microbiota metabolism, oxygen dynamics, and epithelial proliferation. While this study opens new mechanistic avenues, it also underscores the complexity of balancing epithelial growth and homeostasis. Deciphering how HIF1α integrates hypoxic, metabolic, and microbial cues to preserve intestinal equilibrium represents a critical step toward leveraging diet and the microbiome for therapeutic benefit.

## Data Availability

N.A.

## References

[1] Ribeiro Castro P, Corrêa RO, Sérvulo Cruz Angelim MK, de Rezende Rodovalho V, Font Fernandes M, Dias Nirello V, Dos Santos Pereira Gomes AB, de Souza Felipe J, Passarielo Pral L, de Oliveira S, et al. HIF-1 attenuates high-fiber diet-mediated proliferation and stemness of colonic epithelium. Gut Microbes. 2025;17:2543123. doi: 10.1080/19490976.2025.2543123. 40827535 PMC12369635

[2] Kelly CJ, Zheng L, Campbell EL, Saeedi B, Scholz CC, Bayless AJ, Wilson KE, Glover LE, Kominsky DJ, Magnuson A, et al. Crosstalk between microbiota-derived short-chain fatty acids and intestinal epithelial HIF augments tissue barrier function. Cell Host Microbe. 2015;17:662–671. doi: 10.1016/j.chom.2015.03.005. 25865369 PMC4433427

[3] Byndloss MX, Olsan EE, Rivera-Chávez F, Tiffany CR, Cevallos SA, Lokken KL, Torres TP, Byndloss AJ, Faber F, Gao Y, et al. Microbiota-activated PPAR-γ signaling inhibits dysbiotic Enterobacteriaceae expansion. Science. 2017;357:570–575. doi: 10.1126/science.aam9949. 28798125 PMC5642957

[4] Dowdell AS, Roer R, Bhagavatula G, Cartwright IM, Cohen RH, Countess JA, Koch SD, Lee JS, Steiner CA, Thompson NT, et al. Regulation of epithelial HIF by probiotic *Escherichia coli*. bioRxiv. 2025;2025(05):654147.

[5] Kierans SJ, Fagundes RR, Malkov MI, Sparkes R, Dillon ET, Smolenski A, Faber KN, Taylor CT. Hypoxia induces a glycolytic complex in intestinal epithelial cells independent of HIF-1-driven glycolytic gene expression. Proc Natl Acad Sci U S A. 2023;120:e2208117120. doi: 10.1073/pnas.2208117120. 37603756 PMC10469334

[6] Fagundes RR, Bravo-Ruiseco G, Hu S, Kierans SJ, Weersma RK, Taylor CT, Dijkstra G, Harmsen HJM, Faber KN. *Faecalibacterium prausnitzii* promotes intestinal epithelial IL-18 production through activation of the HIF1α pathway. Front Microbiol. 2023;14:1298304. doi: 10.3389/fmicb.2023.1298304. 38163085 PMC10755969

[7] Saeedi BJ, Kao DJ, Kitzenberg DA, Dobrinskikh E, Schwisow KD, Masterson JC, Kendrick AA, Kelly CJ, Bayless AJ, Kominsky DJ, et al. HIF-dependent regulation of claudin-1 is central to intestinal epithelial tight junction integrity. Mol Biol Cell. 2015;26:2252–2262. doi: 10.1091/mbc.E14-07-1194. 25904334 PMC4462943

[8] Michael AK, Stoos L, Crosby P, Eggers N, Nie XY, Makasheva K, Minnich M, Healy KL, Weiss J, Kempf G, et al. Cooperation between bHLH transcription factors and histones for DNA access. Nature. 2023;619:385–393. doi: 10.1038/s41586-023-06282-3. 37407816 PMC10338342

[9] Meng X, Asadi-Asadabad S, Cao S, Song R, Lin Z, Safhi M, Qin Y, Tcheumi Tactoum E, Taudte V, Ekici A, et al. Metabolic rewiring controlled by HIF-1α tunes IgA-producing B-cell differentiation and intestinal inflammation. Cell Mol Immunol. 2025;22:54–67. doi: 10.1038/s41423-024-01233-y. 39543372 PMC11686098

[10] Kaiko GE, Ryu SH, Koues OI, Collins PL, Solnica-Krezel L, Pearce EJ, Pearce EL, Oltz EM, Stappenbeck TS. The colonic crypt protects stem cells from microbiota-derived metabolites. Cell. 2016;165:1708–1720. doi: 10.1016/j.cell.2016.05.018. 27264604 PMC5026192

[11] Kierans SJ, Taylor CT. Regulation of glycolysis by the hypoxia-inducible factor (HIF): implications for cellular physiology. J Physiol. 2021;599:23–37. doi: 10.1113/JP280572. 33006160

[12] Fagundes RR, Taylor CT. Determinants of hypoxia-inducible factor activity in the intestinal mucosa. J Appl Physiol. 2017;123:1328–1334. doi: 10.1152/japplphysiol.00203.2017. 28408694

[13] Fagundes RR, Bourgonje AR, Saeed A, Vich Vila A, Plomp N, Blokzijl T, Sadaghian Sadabad M, von Martels JZH, van Leeuwen SS, Weersma RK, et al. Inulin-grown *Faecalibacterium prausnitzii* cross-feeds fructose to the human intestinal epithelium. Gut Microbes. 2021;13. doi: 10.1080/19490976.2021.1993582 PMC8604389 34793284

[14] Fagundes RR, Belt SC, Bakker BM, Dijkstra G, Harmsen HJM, Faber KN. Beyond butyrate: microbial fiber metabolism supporting colonic epithelial homeostasis. Trends Microbiol. 2024;32:178–189. doi: 10.1016/j.tim.2023.07.014. 37596118

[15] Lee JS, Kao DJ, Worledge CS, Villamaria ZF, Wang RX, Welch NM, Kostelecky RE, Colgan SPE. Coli genetically modified for purine nucleobase release promotes butyrate generation and colonic wound healing during DSS insult. Gut Microbes. 2025;17:2490211. doi: 10.1080/19490976.2025.2490211. 40247632 PMC12013446

[16] Niu B, Li F, Lv X, Xiao Y, Zhu J, Zhao J, Lu W, Chen W. Unveiling the therapeutic potential and mechanism of inulin in DSS-induced colitis mice. Int J Biol Macromol. 2024;280:135861. doi: 10.1016/j.ijbiomac.2024.135861. 39307495

[17] Qiao H, Zhao T, Yin J, Zhang Y, Ran H, Chen S, Wu Z, Zhang R, Wang X, Gan L, et al. Structural characteristics of inulin and microcrystalline cellulose and their effect on ameliorating colitis and altering colonic microbiota in dextran sodium sulfate-induced colitic mice. ACS Omega. 2022;7:10921–10932. doi: 10.1021/acsomega.1c06552. 35415348 PMC8991927

[18] Ariaee A, Koentgen S, Wardill HR, Hold GL, Prestidge CA, Armstrong HK, Joyce P. Prebiotic selection influencing inflammatory bowel disease treatment outcomes: a review of the preclinical and clinical evidence. egastro. 2024;2 [accessed 2025 Nov 4]. https://egastroenterology.bmj.com/content/2/2/e100055. 10.1136/egastro-2023-100055 PMC11731074 39944472

[19] Sheng W, Ji G, Zhang L. Immunomodulatory effects of inulin and its intestinal metabolites. Front Immunol. 2023;14. [accessed 2025 Nov 4]. doi: 10.3389/fimmu.2023.1224092/full. PMC10449545 37638034

[20] Corrêa RO, Castro PR, Fachi JL, Nirello VD, El-Sahhar S, Imada S, Pereira GV, Pral LP, Araújo NVP, Fernandes MF, et al. Inulin diet uncovers complex diet-microbiota-immune cell interactions remodeling the gut epithelium. Microbiome. 2023;11:90. doi: 10.1186/s40168-023-01520-2. 37101209 PMC10131329

[21] He Y, Peng X, Liu Y, Wu Q, Zhou Q, Huang Y, Liu S, Hu L, Fang Z, Lin Y, et al. Long-term maternal intake of inulin exacerbated the intestinal damage and inflammation of offspring rats in a DSS-induced colitis model. Food Funct. 2022;13:4047–4060. doi: 10.1039/D1FO03675K. 35315466

[22] Arifuzzaman M, Won TH, Yano H, Uddin J, Emanuel ER, Hu E, Zhang W, Li T-T, Jin W-B, Grier A, et al. Dietary fiber is a critical determinant of pathologic ILC2 responses and intestinal inflammation. J Exp Med. 2024;221:e20232148. doi: 10.1084/jem.20232148. 38506708 PMC10955042

[23] Miles JP, Zou J, Vijay-Kumar M, Pellizzon M, Ricci M, Gewirtz AT, Chassaing B. Supplementation of low- and high-fat diets with fermentable fiber exacerbates severity of DSS-induced acute colitis. Inflamm Bowel Dis. 2017;23:1133–1143. 28590342 10.1097/MIB.0000000000001155 PMC5497995

[24] Hosono T, Saito K, Arima Y, Ozaki-Masuzawa Y, Seki T. Inulin exacerbates disease severity in a mouse model of ulcerative colitis by causing osmotic diarrhea. Biosci Biotechnol Biochem. 2025;89:1721–1727. doi: 10.1093/bbb/zbaf129. 40905722

[25] Zhao X, Wang Y, Wang Y, Gewirtz AT, Zou J. Inulin aggravates colitis through gut microbiota modulation and MyD88/IL-18 signaling. Gut Microbes. 2025;17:2570425. doi: 10.1080/19490976.2025.2570425. 41133791 PMC12562683

[26] Geertsema S, Fagundes RR, Otten AT, Dijkstra G, Faber KN, Bourgonje AR. Interleukin-18 inhibition in inflammatory bowel disease: a delicate balance. Inflamm Bowel Dis. 2024;30(4):693–694. doi: 10.1093/ibd/izae042. 38442894

[27] Harrison OJ, Srinivasan N, Pott J, Schiering C, Krausgruber T, Ilott NE, Maloy KJ. Epithelial-derived IL-18 regulates Th17 cell differentiation and Foxp3+ treg cell function in the intestine. Mucosal Immunol. 2015;8:1226–1236. doi: 10.1038/mi.2015.13. 25736457 PMC4368110

[28] Levy M, Thaiss CA, Zeevi D, Dohnalová L, Zilberman-Schapira G, Mahdi JA, David E, Savidor A, Korem T, Herzig Y, et al. Microbiota-modulated metabolites shape the intestinal microenvironment by regulating NLRP6 inflammasome signaling. Cell. 2015;163:1428–1443. doi: 10.1016/j.cell.2015.10.048. 26638072 PMC5665753

[29] Guimarães JB, Rodrigues VF, Pereira ÍS, Manso GM da C, Elias-Oliveira J, Leite JA, Waldetario MCGM, de Oliveira S, Gomes ABDSP, Faria AMC, et al. Inulin prebiotic ameliorates type 1 diabetes dictating regulatory T cell homing via CCR4 to pancreatic islets and butyrogenic gut microbiota in murine model. J Leukoc Biol. 2024;115:483–496. doi: 10.1093/jleuko/qiad132. 37947010

